# Characterizing changes in mental health‐related outcomes for health service psychology graduate students during the first year of the COVID‐19 pandemic

**DOI:** 10.1002/jclp.23392

**Published:** 2022-05-24

**Authors:** Katharine E. Daniel, Erica Szkody, Pankhuri Aggarwal, Amy H. Peterman, Jason J. Washburn, Edward A. Selby

**Affiliations:** ^1^ Department of Psychology University of Virginia Charlottesville Virginia USA; ^2^ Department of Psychology Mississippi State University Mississippi State Mississippi USA; ^3^ Department of Psychology Miami University Oxford Ohio USA; ^4^ Department of Psychology University of North Carolina at Charlotte Charlotte North Carolina USA; ^5^ Department of Psychiatry and Behavioral Sciences Northwestern University Chicago Illinois USA; ^6^ Department of Psychology Rutgers University New Brunswick New Jersey USA

**Keywords:** adult mental health, clinical psychology, emotional distress, longitudinal studies, subjective experience

## Abstract

**Objectives:**

Health service psychology (HSP) graduate students experienced adverse mental health outcomes during COVID‐19. However, little is known about how mental health outcomes changed in this population after the onset of COVID‐19.

**Methods:**

*N* = 496 HSP graduate students reported onset or worsening of mental health outcomes, inability to access mental health care, worry about COVID‐19, and stress at two different timepoints during the first year of the COVID‐19 outbreak (timepoint 1: May 1 to June 25, 2020; timepoint 2: September 2 to October 17, 2020). This study tested whether mental health outcomes improved, worsened, or stayed stable during this timeframe. The study also examined whether rising COVID‐19 case rates in the state where a participant lived moderated changes in mental health outcomes.

**Results:**

Overall, HSP graduate students endorsed adverse mental health outcomes at a higher rate during the first survey relative to the second survey. Even still, 62.68% of students reported worsened mental health symptoms, 49.84% reported worsened sleep, and 23.92% reported increased alcohol and substance use in the 2 months leading up to the second survey.

**Conclusion:**

HSP programs should monitor graduate students' evolving mental health, provide wellness resources, and adopt flexible approaches to support graduate students navigating training during periods of immense disruption.

## 1 INTRODUCTION

The coronavirus disease 2019 (COVID‐19) was associated with substantial and prolonged disruptions to daily life (Zhai & Du, 2020) since it was declared a pandemic in March 2020 (World Health Organization, 2020). Early indicators and concerns from experts suggested a wave of mental health problems due to the pandemic (Pfefferbaum & North, 2020), with some populations at greater risk of poor mental health outcomes than others (Purtle, 2020), including graduate students (Chirikov et al., 2020).

Even before the COVID‐19 pandemic, graduate students were 10 times more likely to report symptoms of anxiety, six times more likely to report symptoms of depression, and three times more likely to endorse suicidal ideation than the general population (Borges et al., 2010; Evans et al., 2018; Garcia‐Williams et al., 2014; Kocalevent et al., 2013; Löwe et al., 2008). Specifically, health service psychology (HSP) graduate students disproportionally experienced moderate to severe mental health symptoms, including anxiety (23%) and depression (17%), as well as hazardous alcohol use (10%), suicidal ideation (16%), plans to attempt suicide (4%), and actual suicide attempts during graduate school (0.6%; Hobaica et al., 2021) before COVID‐19. Given that the COVID‐19 pandemic has increased risk for mental illness by exacerbating numerous risk factors (e.g., social isolation; uncertainty, Carleton et al., 2012; financial instability; Hudson, 2005), HSP graduate students' elevated pre‐COVID risk is especially concerning. Indeed, the COVID‐19 pandemic has arguably pushed the United States—including its graduate students—into a mental health crisis (Wan, 2020), or at least has accelerated existing trends. For example, anxiety disorders within the United States were approximately three times more prevalent as of June 2020 compared to the second quarter of 2019 (25.5% vs. 8.1%) and depressive disorders had become approximately four times more prevalent throughout the same time frame (24.3% vs. 6.5%; Czeisler et al., 2020). Among graduate students, the prevalence of major depressive disorder was two times higher and generalized anxiety disorder was 1.5 times higher in 2020 compared to 2019 (Chirikov et al., 2020).

### 1.1 Mental health of hsp graduate students during COVID‐19

Given that HSP graduate students are generally at a greater risk for psychological distress, burnout, and negative mental health outcomes compared to the general population (Myers et al., 2012; Richardson et al., 2020), stressors from the COVID‐19 pandemic are likely to have exacerbated that risk. Further, the unique educational and training requirements of HSP graduate students, specifically their need to provide clinical care while also balancing research and teaching responsibilities (American Psychological Association, 2015), may have placed them at an even greater risk for experiencing negative outcomes. Indeed, 40% of psychology interns and postdoctoral fellows endorsed clinically significant anxiety symptoms and 25% reported clinically significant depressive symptoms within the first 2 months of the then‐apparent US outbreak (Schneider et al., 2021), and these symptoms were most prominent among those who were expected to provide clinical care on‐site versus remotely. Despite the likelihood of increased risk, HSP graduate students may be less able to confidentially establish care with a therapist given that HSP graduate students often have professional conflicts with many of the providers in their schools or community (Hobaica et al., 2021). Taken together, there is ample reason to be concerned for the mental health of HSP graduate students in response to the COVID‐19 pandemic.

### 1.2 Overview and hypotheses

Initial findings suggest that mental health challenges were present for HSP graduate students during the first months of the COVID‐19 outbreak (Schneider et al., 2021). However, previous results have been limited to only those HSP graduate students who had already advanced to or were past their predoctoral internship, and only capture one point in time during the onset of COVID‐19. Given that the COVID‐19 pandemic has been dynamic (e.g., changing information and guidelines from the Centers for Disease Control, fluctuation of mitigation efforts within and between state and local governments and private institutions, peaks rates of positive cases shifting in amount and in geographic location), mental health challenges might also have changed throughout the pandemic. As such, the current study employed a repeated‐measures design to test how mental health‐related indices among *N* = 485 HSP graduate students changed between May 2020 and October 2020 using a two‐wave data collection.

#### 1.2.1 Hypotheses

We sought to examine the rate and change of endorsements of eight binary mental health indicators between two timepoints. Specifically, students indicated whether or not they had experienced each of the following in the 2 months before both survey waves: incidence of suicidal ideation, suicide planning, suicide attempt, and nonsuicidal self‐injury; worsened mental health; worsened sleep; increased usage of alcohol or drugs; and inability to access mental health care treatment or therapy. We also compared level of perceived stress (measured using the Perceived Stress Scale [PSS]; Cohen et al., 1983) and level of COVID‐19 worry (measured using a single 5‐point Likert scale) between the two timepoints. Rather than directional hypotheses, we expected some change, either with mental health‐related indicators improving (as uncertainty reduced and routines became more established) or deteriorating (as burnout increased and the pandemic stretched on).

We also conducted follow‐up, exploratory analyses to examine if changes to these outcomes were moderated by students' year in their HSP training program, given that academic stressors, clinical stressors, and opportunities to establish supportive networks may change over the course of enrollment. We also examined if change in outcomes was moderated by the average number of new COVID‐19 cases confirmed in their state each day over the 2 months before their survey response, assuming that higher rates may pose differential emotional strain or increased mitigation efforts.

Next, we examined the association between inability to access mental health care on subjective stress levels. We expected that students reporting an inability to access mental health treatment due to the COVID‐19 pandemic would report higher levels of concurrent subjective stress. We also expected inability to access mental health care would prospectively predict increased stress levels at the next wave of data collection, even after controlling for concurrent stress.

Finally, we hypothesized that students living in a state with greater numbers of new daily COVID‐19 cases in the 2 months before each survey wave would endorse greater amounts of subjective worry over COVID‐19 and would also report higher levels of subjective stress. Further, we expected that students who endorsed greater amounts of subjective worry over COVID‐19 would also report higher levels of subjective stress. We predicted that these relationships would hold within a given wave of data (i.e., a contemporaneous effect) and we predicted that higher average new daily case rates and increased worry at wave 1 would predict increased stress at the next wave (i.e., a temporal effect).

## 2 METHOD

### 2.1 Procedure

The current study uses data collected by the Council of University Directors of Clinical Psychology (CUDCP) to examine the impact of COVID‐19 on HSP graduate students from May 2020 to March 2021. HSP graduate students pursuing doctoral‐level degrees in clinical, counseling, school, and education psychology were recruited through their DCT, HSP associated listservs, and through participating doctoral programs in the Council of Chairs of Training Councils (CCTC). All participating students provided informed consent before completing online survey measures in a randomized order and all students were treated in accordance with the APA Ethical Code. For the current study, we used data from the first (May 1, 2020, to June 25, 2020) and second (September 2, 2020, to October 17, 2020) waves of the study, both of which occurred before COVID‐19 vaccination distributions (AJMC, 2021). We selected a 12‐week follow‐up after wave 1 to allow for measurement of changes to student mental health indicators and in program training procedures between different semesters. For more information on participant recruitment and procedure, see Boland et al. (2022). The data collection procedures for this study were approved by the Rutgers University IRB.

### 2.2 Participants

A total of 1665 students participated in the first wave of data collection and 646 students participated in the second wave of data collection. Data were linked between both waves of data by the respondent's email address, which limited the number of participants included in the present analyses to only those who provided the same email address at both waves 1 and 2 (*n* = 496). We elected to remove participants lost to follow up, rather than use multiple imputation to retain all first wave survey respondents, because we expect that our pattern of missingness is not at random. These surveys were distributed during a global pandemic and at different times of the academic school year when various yearly milestones and programmatic responsibilities may have been shifting at different times for different programs. Further, we recruited participants through sending requests to directors of clinical training (DCT). As such, participants who completed the first wave of data collection may not have been aware of the second wave if their DCT failed to send out the second request. Given that we did not measure these reasons for systematic missingness, among other potential sources, we do not meet the multiple imputation assumption for missing at random. Given that our data are likely missing not at random, the listwise deletion approach that we took does not introduce additional bias beyond that which would be introduced by using multiple imputation on these data (Arel‐Bundock & Pelc, 2018).^1^ We also removed participants who reported they had already graduated from their program (*n* = 11). This resulted in a final sample of 485 participants. Demographic information for the sample included in the analyses is available in Table 1.

**Table 1:** Demographic characteristics.

Demographic characteristic	Percentage endorsed	
Race and ethnicity^a^		
Non‐Hispanic White	81.00	
Hispanic White	3.71	
Non‐Hispanic Black	3.91	
Hispanic Black	0.21	
Non‐Hispanic Asian	10.10	
Hispanic Asian	0.21	
Non‐Hispanic American Indian or Alaska Native	0.41	
Hispanic American Indian or Alaska Native	0.21	
Non‐Hispanic Middle Eastern or North African	3.30	
Hispanic Middle Eastern or North African	0.41	
Non‐Hispanic Native Hawaiian or Pacific Islander	0.21	
International student (non‐United States)^a^	4.74%	
Self‐reported gender identity^a^		
Man	10.35	
Woman	88.20	
Gender queer, gender nonbinary, or gender nonconforming	2.28	
Transgender man	0.41	
Transgender woman	0.21	
Other gender	0.41	
Geographic region^b^		
Northeast	Wave 1: 22.95	Wave 2: 22.93%
Southeast	Wave 1: 26.53	Wave 2: 26.11%
Northwest	Wave 1: 4.84	Wave 2: 4.46%
Southwest	Wave 1: 24.84	Wave 2: 23.35%
Midwest	Wave 1: 18.32	Wave 2: 21.44%
Mountain West	Wave 1: 2.32	Wave 2: 4.46%

^a^ Percentages are out of *N* = 485 participants.

^b^ Percentages for wave 1 are out of *N* = 475 and percentages for wave 2 are out of *N* = 471. One participant reported being in Hawaii.

### 2.3 Measures

#### 2.3.1 Demographic questions

Students were asked to indicate their age, academic degree type (e.g., PsyD or PhD), academic program area (e.g., clinical psychology, counseling psychology), year in program, race/ethnicity as defined by themselves, gender identity, and current zip code (to track local COVID‐19 case rates).

#### 2.3.2 Epidemic‐pandemic impacts inventory (EPII)

The EPII was designed by Grasso et al. (2020) to examine the impact of the COVID‐19 pandemic on different domains of an individual's personal and family life. Students indicated with a yes/no response whether each item on the inventory applied to them over the last 2 months. This questionnaire was presented at both survey waves. For hypothesis testing, we selected four items directly related to mental health: “Increase in sleep problems or poor sleep quality” to measure worsened sleep; “Increase in use of alcohol or substances” to measure increased alcohol or substance use; “Increase in mental health problems or symptoms (e.g., mood, anxiety, stress)” to measure worsened mental health symptoms; and “Unable to access mental health treatment or therapy” to measure inability access mental health care treatment. In addition to these items, the EPII covers a range of domains that may also be of interest to readers (i.e., work and employment, home life, social activities, economic, emotional health and wellbeing, physical distance and quarantine, infection history, and positive change). As such, we provide those items and their endorsement rates at each survey wave in the Supporting Information.

#### 2.3.3 COVID‐19 worry

Students were asked to rate their level of worry about the coronavirus using the single item: “How worried are you about the coronavirus?” on a scale from 1 (*not at all worried*) to 5 (*extremely worried*). This face‐valid item was presented at both survey waves and was significantly correlated between the two time points (*r* = 0.61, *p* < 0.001).

#### 2.3.4 COVID‐19 rates

The number of positive COVID‐19 cases each day for each state in the United States were obtained from a publicly available data repository (https://covidtracking.com/data/api). We extracted the first difference of COVID‐19 cases each day for the 60 days^2^ leading up to each respondent's survey response, for both survey waves, and for the state where their zip code indicated they were located. The first difference is the amount of change from one time point to the next (i.e., between successive days). As such, each timeseries of 60 difference scores captured the amount of new COVID‐19 cases each day in a given state. Because these data show change across days (the number of *new* cases since the previous day), each timeseries represents the rate of change in new COVID‐19 cases over each 60‐day period. We then averaged the new COVID‐19 case rates within each student for each survey wave such that each average value was derived from 60 days' worth of new COVID‐19 case data. These average first difference scores were used in subsequent analyses.

#### 2.3.5 PSS‐4

The PSS‐4 (Cohen et al., 1983) consists of four items that assess the student's feelings and thoughts over the last 2 months regarding their current level of stress. Students were asked to rate each of the four items on a scale of 1 (*never*) to 5 (*very often*) at both waves of data collection. The validity of the PSS‐4 as a measure of perceived stress has been demonstrated in previous studies (e.g., Warttig et al., 2013). In the current study, the PSS‐4 was used as a measure of current student stress and was collected at both timepoints. Cronbach's *α* was 0.75 at both survey waves.

#### 2.3.6 Self‐care

Students were asked to rate their level of self‐care over the last 2 months using the face‐valid single item: “How would you rate your level of self‐care over the last 2 months?” on a scale of 1 (*no self‐care*) to 5 (*great self‐care*) at both survey ways. Self‐care was significantly correlated between the two survey waves (*r* = 0.58, *p* < 0.001).

#### 2.3.7 Suicide

Students were asked to identify if they had suicidal ideation, made a suicidal plan, attempted suicide, or engaged in nonsuicidal self‐injury since the COVID‐19 pandemic began (at wave 1) or within the 2 months leading up to wave 2. Questions were adapted from the Composite International Diagnostic Interview (CIDI, Robins et al., 1988). Students were asked to indicate yes or no if the following questions applied to them: “Did you feel so low that you thought a lot about committing suicide?,” “Did you make a suicide plan?,” “Did you attempt suicide?,” and “Did you engage in nonsuicidal self‐injury?” We treated these items as separate binary outcomes.

### 2.4 Data analysis

Generalized logistic mixed‐effects models comparing the difference in likelihood for students to endorse eight different binary mental health indicators between both survey waves were conducted using the “glmer” function in the lme4 package in R (Bates et al., 2015). Each binary mental health indicator was modeled as a separate outcome (0 = indicator was not endorsed, 1 = indicator was endorsed). Linear mixed‐effects models comparing changes in COVID‐19 worry, stress, and self‐care over time were modeled with the “lmer” function in the lme4 package in R (Bates et al., 2015). These mixed‐effects models were specified with the survey wave as the fixed effect of interest (0 = first survey wave, 1 = second survey wave) and with a random intercept per student.^3^ Given that there were only two survey waves, we did not model random slopes. We included two demographic control variables in these models to reflect racial minority status (non‐Hispanic White = 0 vs. other = 1) and gender minority status (cis‐gender man = 0 vs. other = 1). For both continuous outcomes and if there was sufficient variation between endorsing and not endorsing a given binary indicator (i.e., the indicator was endorsed between 30% and 70% of the time) we tested for moderation. Specifically, we included the year in one's program and the average first difference of COVID‐19 cases as moderators in two separate follow‐up models. We grand‐mean centered continuous moderators before entering them into the model. We scaled the average first difference of COVID‐19 cases by dividing all values by 1000 so that models would converge. All mixed‐effects models were fit using full information maximum likelihood estimation.

We used structural equation modeling in the lavaan package in R (Rosseel, 2012) to test the association between the inability to access mental health care and self‐reported stress levels. We modeled stress at both survey waves as a latent variable, which resulted in two latent stress variables that were each specified by the four PSS‐4 items from that survey wave. We fixed the path from both latent stress variables to the first PSS‐4 item to 1. We regressed no mental health care access on self‐reported levels of stress within both survey waves. We also regressed no mental health care access at wave 1 on self‐reported stress at wave 2 and self‐reported stress at wave 1 on no mental health care access at wave 2. A path diagram is available in Supporting Information: Figure 1. All regression paths and variance parameters were freely estimated.

Similarly, we used structural equation modeling to test the association between average new COVID‐19 case rates in the 60 days leading up to each survey wave, COVID‐19‐related worry, and self‐reported stress levels. As above, we modeled stress at both survey waves as a latent variable. We regressed the average COVID‐19 new case rate on COVID‐19 worry and on self‐reported levels of stress within both survey waves. We regressed COVID‐19 worry on self‐reported levels of stress within both survey waves. We also regressed average COVID‐19 case rate from wave 1 on COVID‐19 worry and stress at wave 2. Finally, we regressed COVID‐19 worry at wave 1 on stress at wave 2. We centered these average COVID‐19 case rate variables and scaled them to improve model fit by dividing all average values by 1000. A path diagram is available in Supporting Information: Figure 2. All regression paths and variance parameters were freely estimated.

The data that support the findings of this study are available on request from the corresponding author. The data are not publicly available due to privacy or ethical restrictions. Data analysis scripts are openly available at the Open Science Foundation (https://osf.io/rwp6c/?view_only=cb2fbdc6dc64461486850acfa64497c6).

## 3 RESULTS

### 3.1 Differences in mental health outcomes across time

#### 3.1.1 Worry about COVID‐19

After controlling for racial and gender minority status, respondents reported no change in their degree of worry about COVID‐19 between the first (*M* = 3.07, SD = 0.88) and second (*M* = 3.04, SD = 0.80) survey waves (*b* = −0.02, SE = 0.03, *t* = −0.65, *p* = 0.514). The effects of gender and racial minority status were nonsignificant. The interaction between year in program and survey wave was not significant (*b* = −0.03, SE = 0.02, *t* = −1.22, *p* = 0.223). However, students in later years of their program reported a higher degree of COVID‐19 worry than students in earlier years of study (*b* = 0.06, SE = 0.03, *t* = 2.26, *p* < 0.05). Average rate of new local COVID‐19 cases each day for the 2 months before each survey wave was not a significant moderator (*b* = 0.02, SE = 0.02, *z* = 0.689, *p* = 0.492) nor main effect (*b* = −0.02, SE = 0.02, *z* = −0.82, *p* = 0.415) in predicting worry.

#### 3.1.2 Perceived stress

Participants' perceived stress decreased between the first (*M* = 11.56, SD = 2.66) and second (*M* = 11.06, SD = 2.65) waves (*b* = −0.50, SE = 0.11, *t* = −4.49, *p* < 0.001), even after controlling for racial and gender minority status. While the effect of gender minority status on perceived stress was not significant, individuals self‐identifying as belonging to a racial/ethnic minority group reported higher levels of stress, on average, than did non‐Hispanic White individuals (*b* = 0.77, SE = 0.27, *t* = 2.86, *p* < 0.01). Neither the main effect of year in one's program (*b* = −0.05, SE = 0.09, *z* = −0.61, *p* = 0.541) nor its interaction with survey wave (*b* = −0.06, SE = 0.08, *z* = −0.77, *p* = 0.444) were significant. Average rate of new local COVID‐19 cases each day for the 2 months before each survey wave was also not a significant moderator (*b* = −0.06, SE = 0.08, *z* = −0.82, *p* = 0.411) nor main effect (*b* = 0.09, SE = 0.07, *z* = 1.26, *p* = 0.207) in predicting perceived stress.

#### 3.1.3 Worsening of mental health symptoms

72.37% of respondents endorsed worsening mental health symptoms during the 2 months before the first survey wave and 62.68% endorsed worsening mental health symptoms during the 2 months before the second survey wave. Students were less likely to endorse worsening mental health symptoms at the second wave of data collection compared to the first (*b* = −0.63, SE = 0.17, *z* = −3.67, *p* < 0.001), even after controlling for the nonsignificant effects of gender minority status and racial minority status. Neither the main effect of year in one's program (*b* = −0.05, SE = 0.10, *z* = −0.53, *p* = 0.598) nor its interaction with survey wave (*b* = −0.19, SE = 0.12, *z* = −1.60, *p* = 0.110) were significant. Average rate of new local COVID‐19 cases each day for the 2 months before each survey wave was also not a significant moderator (*b* = 0.12, SE = 0.11, *z* = 1.15, *p* = 0.252) nor main effect (*b* = −0.06, SE = 0.09, *z* = −0.65, *p* = 0.519) in predicting worsening of mental health symptoms.

Examination of different trajectories of change indicate that 17.94% of students never endorsed worsening mental health symptoms, 19.38% of students endorsed worsening mental health symptoms at wave 1 but not at wave 2, 9.69% of students endorsed worsening mental health symptoms at wave 2 but not at wave 1, and 52.99% of students endorsed worsening mental health symptoms at both waves. This suggests that over half of the HSP graduate students included in this sample may have experienced increasingly worsening mental health symptoms across the first year of the COVID‐19 pandemic.

#### 3.1.4 Increase in trouble sleeping

67.01% of respondents endorsed worsening sleep during the 2 months before the first survey wave and 49.84% endorsed worsening sleep during the 2 months before the second survey wave. After controlling for gender and racial minority status, students were less likely to endorse worsening sleep at the second wave of data collection compared to the first (*b* = −1.08, SE = 0.18, *z* = −6.11, *p* < 0.001). Students who identified as belonging to a racial/ethnic minority group were more likely to endorse trouble sleeping, on average, than participants who identified as being non‐Hispanic White (*b* = 0.83, SE = 0.29, *z* = 2.86, *p* < 0.01). The effect of gender minority status was not significant. Neither the main effect of year in one's program (*b* = 0.02, SE = 0.10, *z* = 0.22, *p* = 0.823) nor its interaction with survey wave (*b* = −0.11, SE = 0.12, *z* = −0.98, *p* = 0.329) were significant. Average rate of new local COVID‐19 cases each day for the 2 months before each survey wave was also not a significant moderator (*b* = 0.05, SE = 0.10, *z* = 0.54, *p* = 0.588) nor main effect (*b* = −0.001, SE = 0.09, *z* = −0.01, *p* = 0.989) in predicting worsening sleep.

Regarding the different trajectories of change, 25.36% of students never endorsed worsening sleep, 25.15% of students endorsed worsening sleep at wave 1 but not at wave 2, 7.62% of students endorsed worsening sleep at wave 2 but not at wave 1, and 41.86% of students endorsed worsening sleep at both waves. This suggests that only a quarter of the HSP graduate students included in this sample reported no adverse change to their quality of sleep during the first year of the COVID‐19 pandemic. Notably, due to the binary nature of this response scale, we are unable to learn whether those who reported stable sleep across both waves were initially satisfied with their quality of sleep.

#### 3.1.5 Increase in using alcohol or drugs

35.05% of respondents endorsed increased alcohol or drug use during the 2 months before the first survey wave and 23.92% endorsed increased alcohol or drug use during the 2 months before the second survey wave. After controlling for gender and racial minority status, students were less likely to endorse increased use of alcohol or drugs at the second wave of data collection compared to the first (*b* =−1.06, SE = 0.22, *z* = −4.83, *p* < 0.001). Students who reported holding a marginalized gender identity were less likely to report increased use of alcohol or drugs, on average, than those who identified as cis‐gendered men (*b* = −1.21, SE = 0.60, *z* = −2.01, *p* < 0.05). The effect of racial minority status was not significant. Neither the main effect of year in one's program (*b* = 0.05, SE = 0.14, *z* = 0.38, *p* = 0.701) nor its interaction with survey wave (*b* = 0.05, SE = 0.15, *z* = 0.37, *p* = 0.710) were significant. Average rate of new local COVID‐19 cases each day for the 2 months before each survey wave was also not a significant moderator (*b* = 0.14, SE = 0.14, *z* = 0.99, *p* = 0.322) nor main effect (*b* = −0.14, SE = 0.13, *z* = −1.09, *p* = 0.277) in predicting increased alcohol or drug use.

Regarding the different trajectories of change, 59.59% of students never endorsed increased substance use, 16.49% of students endorsed increased substance use at wave 1 but not at wave 2, 5.36% of students endorsed increased substance use at wave 2 but not at wave 1, and 18.56% of students endorsed increased substance use at both waves. Due to the measurement approach, we are unable to learn whether the 16.49% of students who said their drinking or drug use increased at wave 1 but not at wave 2 reduced their substance use over time or if they continued to use substances at a stable rate following their initial increase.

#### 3.1.6 Nonsuicidal self injury

3.94% of respondents endorsed nonsuicidal selfinjury (NSSI) during the 2 months before the first survey wave and 3.20% endorsed NSSI during the 2 months before the second survey wave. There was no difference in the likelihood for students to report engaging in nonsuicidal self‐injury between the first two waves (*b* =−0.90, SE = 0.79, *z* = −1.14, *p* = 0.255). The effects of gender and racial minority status were both nonsignificant.

#### 3.1.7 Suicidal ideation

8.71% of respondents endorsed suicidal ideation (SI) during the 2 months before the first survey wave and 6.87% endorsed SI during the 2 months before the second survey wave. Students were less likely to endorse suicidal ideation in the 2 months leading up to the second wave of data collection compared to the time before the first wave (*b* = −1.07, SE = 0.54, *z* = −1.98, *p* < 0.05). The effects of gender and racial minority status were both nonsignificant.

#### 3.1.8 Suicide plan

1.03% of respondents planned for suicide during the 2 months before the first survey wave and 0.64% planned for suicide during the 2 months before the second survey wave. Given that planning for suicide was reported so infrequently, we did not conduct significance tests comparing endorsement rates across survey waves.

#### 3.1.9 Suicide attempt

0.41% of respondents attempted suicide during the 2 months before the first survey wave and 0% attempted suicide during the 2 months before the second survey wave. Given that attempting suicide was also reported so infrequently, we did not conduct significance tests comparing endorsement rates across survey waves.

#### 3.1.10 Unable to access mental health services

8.25% of respondents reported being unable to access mental health services during the 2 months before the first survey wave and 7.84% reported no access during the 2 months before the second survey wave. There was no difference in the likelihood for students to report being unable to access mental health services between the first two survey waves (*b* = −0.17, SE = 0.42, *z* = −0.42, *p* = 0.676). The effects of racial and gender minority status were both nonsignificant.

Regarding the different trajectories of change, 86.80% of students never endorsed being unable to access mental health care, 5.36% of students endorsed lacking access at wave 1 but not at wave 2, 4.95% of students endorsed lacking access at wave 2 but not at wave 1, and 2.89% of students endorsed being unable to access mental health care at both waves.

### 3.2 Lack of access to mental health services predicting levels of stress

The first SEM model demonstrated good fit to the data (*χ* ^2^(31) = 84.17, *p* < 0.001, comparitive fit index (CFI) = 0.956, root mean square error of approximation (RMSEA) = 0.060, standardized root mean squared residual (SRMR) = 0.032; Hu & Bentler, 1999). As hypothesized, reporting a lack of access to mental health services at the first wave of data collection predicted higher levels of perceived stress within the same wave (*ß* = 0.15, 95% CI = [0.10, 0.52], *p* < 0.01). However, inconsistent with hypotheses, this relationship was not observed within the second wave of data (*ß* = −0.003, 95% CI = [−0.17, 0.16], *p* = 0.947). Further, endorsing a lack of access to mental health services at wave 1 did not significantly predict perceived stress at wave 2 (*ß *= 0.60, 95% CI = [−0.05, 0.28], *p* = 0.180). That said, higher levels of perceived stress at wave 1 predicted being significantly more likely to report having no access to mental health care services at wave 2 (*ß* = 0.10, 95% CI = [0.002, 0.10], *p* < 0.05). Full statistics are provided in Supporting Information: Table 1.

### 3.3 New COVID‐19 cases, COVID‐19 worry, and levels of stress

The second SEM model also showed good fit to the data (*χ* ^2^(46) = 108.538, *p* < 0.001, CFI = 0.951, RMSEA = 0.056, SRMR = 0.040; Hu & Bentler, 1999). As hypothesized, reporting greater levels of worry about COVID‐19 predicted significantly greater levels of perceived stress, both within wave 1 (*ß* =0.18, 95% CI = [0.04, 0.18], *p* < 0.01) and within wave 2 (ß = 0.15, 95% CI = [0.03, 0.16], *p* < 0.01). Further, greater levels of COVID‐19 worry at wave 1 predicted significantly higher levels of COVID‐19 worry at wave 2 (*ß* = 0.61, 95% CI = [0.48, 0.61], *p* < 0.001). Similarly, greater levels of stress at wave 1 predicted significantly higher levels of stress at wave 2 (*ß* = 0.74, 95% CI = [0.50, 0.84], *p* < 0.001). However, inconsistent with hypotheses, average new daily COVID‐19 cases were not significantly associated with COVID‐19 worry or with levels of perceived stress, either contemporaneously or prospectively (all *p*s > 0.104). Full statistics are provided in Supporting Information: Table 2.

### 3.4 Follow‐up analysis

Given that there was some evidence of improved outcomes over time, we decided to test if respondents were increasing their use of self‐care as one potential explanation for this pattern of results. However, controlling for gender and racial minority status, respondents did not report a change in the quality of their self‐care between the first (*M* = 3.20, SD = 0.88) and second (*M* = 3.22, SD = 0.83) survey waves (*b* = 0.03, SE = 0.04, *t* = 0.74, *p* = 0.458). Students who identified as belonging to a racial/ethnic minority group reported lower levels of self‐care, on average, than did non‐Hispanic White respondents (*b* = −0.21, SE = 0.09, *t* = −2.21, *p* < 0.05). Identifying as a gender minority was not associated with self‐reported level of self‐care.

## 4 DISCUSSION

In the current study, we examined the change in mental health‐related indices among HSP graduate students across two timepoints within the first year of the COVID‐19 pandemic. Our results indicated that a smaller proportion of students enrolled in HSP graduate courses tended to report onset or worsening of mental health outcomes, difficulty sleeping, use of alcohol and other substances, suicidal ideation, and NSSI as the pandemic progressed during the first year. However, 52.99% of students endorsed worsening mental health symptoms at both waves, 41.86% of students endorsed worsening sleep at both waves, and 18.56% of students endorsed increased substance use at both waves, suggesting that a sizable number of HSP graduate students were deteriorating over the first year of the COVID‐19 pandemic. While health‐related outcomes among HSP graduate students have received some scholarly attention, our study is the first to highlight different mental health outcomes in this group between two timepoints during a global pandemic. The present study provides valuable insights into the mental health progression of HSP graduate students during a worldwide pandemic, highlighting specific areas of intervention that programs and institutions can consider.

While students endorsed a range of stressors in the context of the COVID‐19 pandemic, fewer HSP graduate students reported adverse mental health outcomes throughout Fall 2020 compared to Summer 2020 (although we observed no differences between the two survey waves on likelihood to engage in NSSI, practice of self‐care, or ability to access mental health services). However, because we used binary measures to assess worsening of mental health symptoms, sleep, and substance use, we are not positioned to differentiate between participants who endorsed these indicators at wave 1 and not at wave 2 because their symptoms improved over time from those whose symptoms remained stable, albeit elevated, in the months following wave 1. This measurement ambiguity notwithstanding, there are many possible explanations for why endorsement rates tended to decrease, on average, across the two survey waves. Although we are not positioned to test all possibilities, some plausible explanations may be due to students acquiring new skills, seeking/obtaining various forms of support, and benefiting from improved communication from their university and from greater certainty in the information provided around COVID‐19 such that students felt increasingly prepared to face the challenges, stress, and worries associated with COVID‐19. Indeed, US college students reported seeking support from others and helping themselves by using a range of coping mechanisms (e.g., self‐isolating, disconnecting from media, meditating, breathing exercises, and keeping routines; Son et al., 2020). Moreover, students may have experienced differences in academic and social obligations over this timeframe (e.g., fewer deadlines for academic milestones, return from summer break, or rejuvenating time spent away from the office/classroom). Further, given that HSP graduate students continued to offer clinical services throughout the pandemic, they may have provided psychoeducation to their clients around how to best manage pandemic‐related difficulties, which ultimately may have been advantageous to them in their personal life. That said, a sizable percentage of students continued to endorse some degree of psychological distress at the second survey wave.

Our results are consistent with other investigations that have highlighted the negative impact of COVID‐19 on students. For example, in a study conducted with medical students in China, 24.9% of students reported experiencing at least mild levels of anxiety at some point during the COVID‐19 pandemic (Cao et al., 2020). Similarly, 71% of the students at a public university in the United States indicated increased levels of stress and anxiety due to the global pandemic (Son et al., 2020). Further, there is some evidence to suggest that the emotional burden of the COVID‐19 pandemic was more severe for students compared to the general population (Duong et al., 2020). Given that HSP graduate students have been shown to have high levels of self‐reported stress, suicidal ideation, and burnout even before the COVID‐19 pandemic (e.g., Hobaica et al., 2021; Richardson et al., 2020) and students appear to be at unique risk for COVID‐19‐related stressors, our findings suggest that the pandemic may have exacerbated a pre‐existing risk for psychological difficulties among HSP graduate students during their training, and this risk may have compounded overtime for some students (i.e., over half of the HSP graduate students included in this sample experienced increasingly worsening mental health symptoms across the first year of the COVID‐19 pandemic).

### 4.1 Largely null moderation effects for year in program and average new COVID cases

Students in later years of training reported higher levels of COVID‐19 worry, possibly because senior students may have been more likely continue working at in‐person external practica where risk of exposure was higher (Schneider et al., 2021). It is interesting to note that year in one's training program did not significantly predict or moderate any other outcome, despite each year being associated with different milestones (e.g., dissertation, comprehensive examination) and unique demands (e.g., research assistantship, external traineeship). It is possible that the general stress from COVID‐19 was not demand‐specific. Notably, however, students who were further along in their program were more likely to be excluded from analyses due to a failure to return to the second wave. As such, it may be that these null results are in part driven by bias inherent in our sample, particularly if more senior students chose not to participate due to either more or less demands associated with their year in program.

Average COVID‐19 new case rates was also not a significant predictor or moderator of any outcome. Although we expected increasing COVID‐19 cases local to where a participant lived would exacerbate mental health outcomes, it is possible that different points in time along any given COVID‐19 case rate surge drove similar mental health outcomes, but for different reasons. For example, before a surge in cases, one might reasonably worry about a future surge. During a surge, one might reasonably worry about immediate risk to health and disruptions to personal demands and responsibilities. Following a surge, one might reasonably worry about the consequences of a previous surge or the threat of future surges. These findings suggest little impact on fluctuations in COVID‐19 cases and mental health outcomes. However, more research to this point is needed, especially with studies that do not need to aggregate fluctuating COVID cases in the same manner as the current study.

### 4.2 Limitations and future directions

While the current study advanced the literature concerning mental health and stressors faced by HSP graduate students during the beginning stages of the COVID‐19 pandemic, several limitations of the study should be considered. First, despite recruiting from a large pool of HSP graduate students across several disciplines at two timepoints, there was a large rate of attrition between the two survey waves. To closely examine changes in COVID‐19‐related mental health outcomes for HSP graduate students between two timepoints (a strength of this study), we decided to include only those participants who completed both survey waves. This decision reduced our sample size, which may have negatively affected our ability to detect change on low base rate events (e.g., suicide attempts). Further, there were several demographic differences between those who did not return for the second wave of data collection (and were therefore excluded from analyses) and those who were included in analyses. Although, attrition was not predicted by COVID‐19 related worry or stress, participants retained in analyses were significantly more likely to be in a PhD program, be a member of a racial/ethnic minority group, be younger, and be at an earlier stage of their program, indicating a potential bias in the complete case sample.

Second, while the study sample was largely representative of HSP overall, the sample was not as diverse as expected, which may have limited our ability to capture the experiences of minority students. This may be especially true given the unique stressors faced by minority students in the United States during the first year of the COVID‐19 pandemic. Indeed, studies have found that racial and ethnic minorities faced additional challenges because of systemic disparities in healthcare and the workforce (Bureau of Labor Statistics, 2020; Siders & Gerber‐Chavez, 2021). These challenges were further compounded by increases in racism and discrimination as the nation navigated protests and social uprisings following the death of George Floyd (Liu & Modir, 2020; Tavernise & Oppel, 2020). To this end, we did find that individuals self‐identifying as belonging to a racial/ethnic minority group reported higher levels of perceived stress, were more likely to endorse trouble sleeping, and reported lower levels of self‐care than did non‐Hispanic White respondents, on average, across both survey waves. However, due to the scope of this manuscript, these lived experiences could not be evaluated in more detail.

Third, while this study helps us understand the different COVID‐19 related mental health outcomes of HSP graduate students over a span of approximately 7 months, it is limited in its ability to capture more distal and long‐lasting impacts for this group (e.g., securing an internship or postdoctoral position, employment outcomes, licensing rates, citation index). Relatedly, by having only two timepoints, we were unable to assess more nuanced trajectories of mental health outcomes. For example, given regional fluctuations in the pandemic over time, it is possible that a longitudinal study spanning a longer period of time with more frequent assessment points could help better map the changes in students' mental health and pave the way for more effective interventions.

Fourth, many of our outcomes were measured by asking participants to report if they had experienced a change to some mental health‐related outcome over the 2 months before each survey wave (e.g., “Increase in mental health problems or symptoms [e.g., mood, anxiety, stress]”). By examining perceived “increases” in mental health problems using binary indicators, it is possible that a student who endorsed an increase in mental health symptoms during wave 1 may not have endorsed an increase in mental health symptoms at wave 2, even if their absolute level of mental health symptoms did not change between the two survey waves. As such, our results suggest that the outcomes we tested did not continue to worsen further for all students who initially reported an increase in symptoms, but we cannot say that they necessarily improved. That said, having evidence to suggest that mental health outcomes did not continue to exponentially worsen for all students who initially reported worsening symptoms is useful when characterizing mental health outcomes for HSP graduate students during the first year of the COVID‐19 pandemic.

Fifth, to minimize burden on students during an already incredibly challenging time, while still capturing valuable data on a range of mental health constructs potentially directly or indirectly affected by the pandemic, we limited the number of items included in our surveys. While practical, this decision does limit our ability to investigate validity and reliability of the single item indicators included in our study.

### 4.3 Recommendations for programs and institutions

#### 4.3.1 Improve support systems to alleviate stress

A major stressor experienced by many students during the first year of the COVID‐19 pandemic was feelings of isolation. HSP graduate students may benefit from an increase in social support systems at the institutional and individual level to help them feel more connected to the campus atmosphere and to their peers. Institutions can help promote social support by providing opportunities for student engagement in virtual formats when in‐person formats are unsuitable. For example, institutions can develop support groups for students to share their coping strategies, talk about mental health, and support each other through their educational training (e.g., Zhai & Du, 2020). Graduate HSP institutions may also consider adopting a mentorship model to provide additional supports for students at all stages of their academic career. In support of this approach, studies have shown that students benefit from being paired with senior students, students from similar demographic populations, and across programs to reduce stress and improve mental health outcomes (Albott et al., 2020; Kazerooni et al., 2020).

#### 4.3.2 Offer mental health first aid interventions

HSP graduate students' mental health problems have escalated during the pandemic; however, many students may not have access to mental health services due to the high demand on those resources, the cost of pursuing individual therapy, and the time required to attend services (Wasil et al., 2021). Digital single session interventions may help to bridge the gap between HSP graduate students' need for services and access to care (Wasil et al., 2021). Individuals can access these digital tools and complete evidence‐based self‐help interventions. These digital tools are a cost‐effective, time‐effective intervention that can rapidly reach students in need and may be helpful in times of crises. It is important not to assume that HSP graduate students are provided psychoeducational tools about coping with stress and worry, tips for living a healthy lifestyle, and warning signs surrounding mental health simply because of their area of study. Students may continue to benefit from receiving tools and guides from their institutions to have on hand when needed (Zapata‐Ospina et al., 2021). In addition, students may benefit from broad screenings to assess that their basic mental health and technology needs are being met. These screeners can help identify students who may be at risk and in need of more support.

### 4.4 Conclusion and call for continued action

We found that fewer HSP graduate students reported worsening mental health outcomes (i.e., worsened mental health symptoms, worsened sleep, increased substance use, experience of suicidal ideation) at the second survey wave compared to the first. Further, participants reported lower levels of perceived stress over the first year of the COVID‐19 pandemic, despite worry about COVID‐19 and levels of self‐care remaining stable during this timeframe. Moreover, living in a state with relatively higher increases in COVID‐19 cases or being at different stages of training were not associated with changes in these outcomes over time. However, of the 485 HSP graduate students included in analyses, only 17.94% never endorsed worsening mental health symptoms, 25.15% never endorsed worsening sleep, and 59.59% never endorsed increased substance use across both survey waves, although our data cannot contextualize the severity of these individuals' mental health symptoms, quality of sleep, or rates of substance use. Despite some ambiguity inherent in these data, results motivate a call to action for programs and institutions to provide emotional and instrumental support during the pandemic and beyond to help HSP graduate students cope with their continued exposure to COVID‐19 stressors. Further, given that the span of time captured by these data is short, it will be important to continue to evaluate the unique pandemic stressors that arise in the future for HSP graduate students.

## CONFLICTS OF INTEREST

The authors declare no conflicts of interest.

### 1 DATA AVAILABILITY STATEMENT

The data that support the findings of this study are available on request from the corresponding author. The data are not publicly available due to privacy or ethical restrictions.

### 1 PEER REVIEW

The peer review history for this article is available at https://publons.com/publon/10.1002/jclp.23392

## Supporting information

Supporting information.

## ENDNOTES

1 Our decision to use listwise deletion and expectation that our data were missing not at random does introduce bias insofar as to whom our results may generalize. To transparently characterize who our findings may best generalize to, we conducted a series of comparisons between participants who were excluded from analyses and those that were included. While these analyses do not speak to unmeasured patterns in missingness, we offer them up front to contextualize our final sample as best as possible. Compared to those who were excluded from analyses for not returning to the second wave of data collection, participants retained in analyses were significantly more likely to be in a PhD program (*t*(1440) = 3.66, *p* < 0.001), be a member of a racial/ethnic minority group (*t*(1440) = 5.63, *p* < 0.001), be younger (*t*(1440) = 3.86, *p* < 0.001), and be at an earlier stage of their program (*t*(1086.83) = 1.99, *p* < 0.05). There were no differences in gender, COVID‐19 worry, or stress between participants who completed both waves of data collection and those who dropped out before the second wave.

2 We chose 60 days because this approximates 2 months, which is consistent with the timeframe we asked respondents to reflect over for trait questionnaires.

3 We attempted to include participant's state as a random effect given that participants' COVID‐related mental health experiences may have differed as a function of where they were living. However, these models did not converge because many states were too infrequently represented in our data. Final models did not include a random effect for state.
