# Supplementary material for: Characterizing changes in mental health‐related outcomes for health service psychology graduate students during the first year of the COVID‐19 pandemic
Source: J Clin Psychol. 2022 May 24;78(11):2281–98. doi: 10.1002/jclp.23392 (PMC9347532; doi:10.1002/jclp.23392)
Supplement: Supplementary file 1 — Supporting information. [file JCLP-78-2281-s001.docx]

**Characterizing Changes in Mental Health-Related Outcomes for Health Service Psychology Graduate Students within the First Year of the COVID-19 Pandemic**

**SUPPLEMENTAL MATERIALS**

**Supplemental Figures**


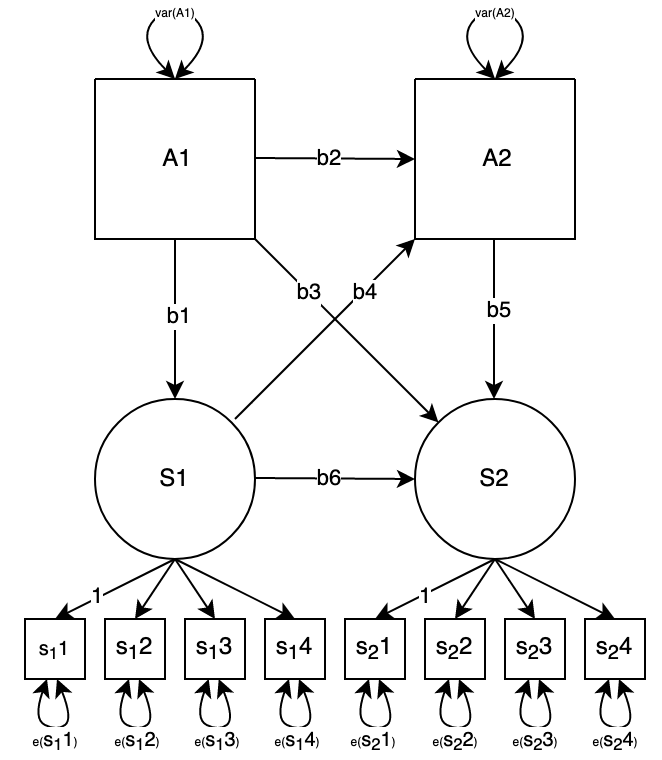


**Figure S1.** Path diagram testing the relationship between lack of access to therapy and perceived stress. A1 = lack of access to therapy at survey wave one. A2 = lack of access to therapy at survey wave two. S1 = perceived stress at survey wave one, where S1 is a latent variable predicting each of the four perceived stress scale items administered at that survey wave (s_1_1-s_1_4). S2 = perceived stress at survey wave two, where S2 is a latent variable predicting each of the four perceived stress scale items administered at that survey wave (s_2_1-s_2_4).


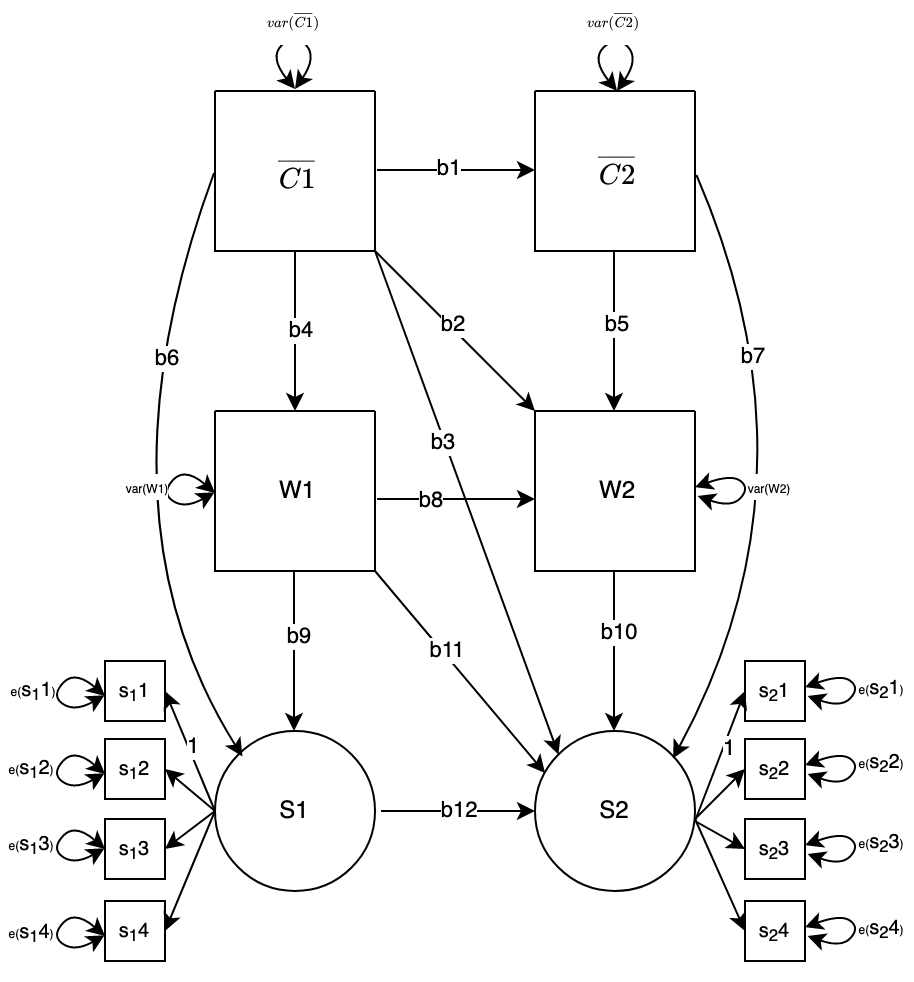


**Figure S2.** Path diagram testing the relationship between average new positive COVID-19 case rate, COVID-19-related worry, and perceived stress. C1 = Average new daily COVID-19 case rate over the 60 days leading up to wave one in the state in which the respondent was located. C2 = Average new daily COVID-19 case rate over the 60 days leading up to wave two in the state in which the respondent was located. W1 = level of COVID-19-related worry at wave one. W2 = level of COVID-19-related worry at wave two. S1 = perceived stress at survey wave one, where S1 is a latent variable predicting each of the four perceived stress scale items administered at that survey wave (s_1_1-s_1_4). S2 = perceived stress at survey wave two, where S2 is a latent variable predicting each of the four perceived stress scale items administered at that survey wave (s_2_1-s_2_4).

**Table S1**

*Association between Lack of Access to Mental Health Services and Levels of Stress*

| **Parameter** | **Standardized Estimate** | **95% CI Lower** | **95% CI Upper** | ***p*** |
| --- | --- | --- | --- | --- |
| **b1** | 0.15 | 0.10 | 0.52 | .004 |
| **b2** | 0.39 | 0.23 | 0.38 | < .001 |
| **b3** | 0.60 | -0.05 | 0.28 | .180 |
| **b4** | 0.10 | 0.002 | 0.10 | .040 |
| **b5** | -0.003 | -0.17 | 0.16 | 0.947 |
| **b6** | 0.74 | 0.51 | 0.83 | < .001 |
| **var(A1)** | 1.00 | 0.06 | 0.08 | < .001 |
| **var(A2)** | 0.89 | 0.06 | 0.07 | < .001 |
| **e(s_1_1)** | 0.66 | 0.50 | 0.67 | < .001 |
| **e(s_1_2)** | 0.44 | 0.21 | 0.30 | < .001 |
| **e(s_1_3)** | 0.68 | 0.32 | 0.43 | < .001 |
| **e(s_1_4)** | 0.43 | 0.38 | 0.55 | < .001 |
| **e(s_2_1)** | 0.71 | 0.53 | 0.71 | < .001 |
| **e(s_2_2)** | 0.46 | 0.23 | 0.33 | < .001 |
| **e(s_2_3)** | 0.61 | 0.32 | 0.43 | < .001 |
| **e(s_2_4)** | 0.43 | 0.37 | 0.54 | < .001 |

*Note.* See Figure 1 path diagram to interpret parameters.

**Table S2**

*Association between Average New Daily COVID-19 Case Rate, COVID-19 Worry, and Levels of Stress*

| **Parameter** | **Standardized Estimate** | **95% CI Lower** | **95% CI Upper** | ***p*** |
| --- | --- | --- | --- | --- |
| **b1** | 0.02 | -0.14 | 0.20 | 0.701 |
| **b2** | -.04 | -.06 | 0.20 | 0.295 |
| **b3** | -0.03 | -.04 | 0.02 | 0.481 |
| **b4** | -0.08 | -0.10 | -.01 | 0.104 |
| **b5** | -0.01 | -0.02 | 0.02 | 0.842 |
| **b6** | 0.05 | -0.02 | 0.06 | 0.335 |
| **b7** | 0.04 | -0.01 | 0.02 | 0.423 |
| **b8** | 0.61 | 0.48 | 0.61 | < .001 |
| **b9** | 0.18 | 0.04 | 0.18 | < .01 |
| **b10** | 0.15 | 0.03 | 0.16 | < .01 |
| **b11** | 0.02 | -0.05 | 0.07 | 0.789 |
| **b12** | 0.74 | 0.50 | 0.84 | < .001 |
| **var(C1)** | 1.00 | 2.04 | 2.66 | < .001 |
| **var(C2)** | 1.00 | 6.57 | 8.52 | < .001 |
| **var(W1)** | 0.99 | 0.69 | 0.90 | < .001 |
| **var(W2)** | 0.63 | 0.35 | 0.46 | < .001 |
| **e(s_1_1)** | 0.67 | 0.51 | 0.70 | < .001 |
| **e(s_1_2)** | 0.44 | 0.21 | 0.30 | < .001 |
| **e(s_1_3)** | 0.67 | 0.32 | 0.44 | < .001 |
| **e(s_1_4)** | 0.44 | 0.39 | 0.57 | < .001 |
| **e(s_2_1)** | 0.72 | 0.54 | 0.73 | < .001 |
| **e(s_2_2)** | 0.48 | 0.25 | 0.36 | < .001 |
| **e(s_2_3)** | 0.61 | 0.31 | 0.42 | < .001 |
| **e(s_2_4)** | 0.44 | 0.34 | 0.55 | < .001 |

*Note.* See Figure 2 path diagram to interpret parameters.

To further characterize changes in HSP students’ experiences in the early months of the COVID-19 Pandemic, we selected a set of EPII items that HSP students and directors of clinical training representing seven different CUDCP programs expected would capture aspects of mental health relevant to HSP students. We characterized the frequency with which each of these stressors was endorsed within the sample at both survey waves. Stressors include those related to physical distancing, quarantine, and infection history (Table S3); work, employment, education, and training (Table S4); financial stability, emotional health, and well-being (Table S5); and home life (Table S6). In general, endorsement rates appear to either be stable between both survey waves or become less frequently endorsed over time across these stressor domains. However, we did not run significance tests on these items given that 64 additional tests would have increased the false discovery rate from these data. That said, we encourage researchers to run secondary data analyses on any items of notable interest from the data counts provided below.

**Table S3**

*Direct Effects of COVID-19*

|  | Wave 1 | | Wave 2 | |
| --- | --- | --- | --- | --- |
| Physical Distancing and Quarantine | % | n | % | n |
| Isolated or quarantined due to possible exposure to this disease. | 34.27 | 170 | 24.80 | 123 |
| Isolated or quarantined due to symptoms of this disease. | 9.07 | 45 | 9.68 | 48 |
| Isolated due to existing health conditions that increase risk of infection or disease. | 15.52 | 77 | 10.89 | 54 |
| Limited physical closeness with child or loved one due to concerns of infection. | 24.80 | 123 | 17.54 | 87 |
| Moved out or lived away from family due to a high-risk job (e.g., health care worker, first responder). | 3.02 | 15 | 0.60 | 3 |
| Close family member not in the home was quarantined. | 8.67 | 43 | 9.07 | 45 |
| Family member was unable to return home due to quarantine or travel restrictions. | 6.85 | 34 | 5.85 | 29 |
| Entire household was quarantined for a week or longer. | 21.17 | 105 | 7.66 | 38 |
| Infection History | % | n | % | n |
| Currently have symptoms of this disease but have not best tested. | 0.81 | 4 | 0.40 | 2 |
| Tested and currently have this disease. | 0.00 | 0 | 0.20 | 1 |
| Had symptoms of this disease but never tested. | 12.70 | 63 | 5.65 | 28 |
| Tested positive for this disease but no longer have it. | 0.60 | 3 | 1.61 | 8 |
| Got medical treatment due to severe symptoms of this disease. | 1.21 | 6 | 0.81 | 4 |
| Hospital stay due to this disease. | 0.00 | 0 | 0.00 | 0 |
| Someone died of this disease while in our home | 0.00 | 0 | 0.00 | 0 |
| Death of close friend or family member from this disease. | 3.43 | 17 | 2.82 | 14 |

*Note. n* = the number of participants who endorsed each variable. % = the percentage of participants that endorsed each variable.

**Table S4**

*COVID-19 stressors experienced during graduate school.*

|  | Wave 1 | | Wave 2 | |
| --- | --- | --- | --- | --- |
| Work and Employment | % | n | % | n |
| Laid off from job or had to close own business. | 4.64 | 23 | 2.02 | 10 |
| Reduced work hours or furloughed. | 19.76 | 98 | 13.71 | 60 |
| Had to continue to work even though in close contact with people who might be infected (e.g., customers, patients, co-workers). | 14.31 | 71 | 28.83 | 143 |
| Spend a lot of time disinfecting at home due to close contact with people who might be infected at work. | 19.76 | 98 | 19.56 | 97 |
| Increase in workload or work responsibilities. | 40.52 | 201 | 36.90 | 183 |
| Hard time doing job well because of needing to take care of people in the home. | 13.71 | 68 | 8.67 | 43 |
| Hard time making the transition to working from home. | 63.71 | 316 | 48.39 | 240 |
| Provided direct care to people with the disease (e.g., doctor, nurse, patient care assistant, radiologist). | 2.02 | 10 | 1.61 | 8 |
| Provided supportive care to people with the disease (e.g., medical support staff, custodial, administration). | 6.25 | 31 | 4.23 | 21 |
| Provided care to people who died as a result of the disease. | 0.81 | 4 | 0.40 | 2 |
| Education and Training | % | n | % | n |
| Had a child in home who could not go to school. | 4.64 | 23 | 4.03 | 20 |
| Adult unable to go to school or training for weeks or had to withdraw. | 26.41 | 131 | 10.48 | 52 |

*Note. n* = the number of participants who endorsed each variable. % = the percentage of participants that endorsed each variable.

**Table S5**

*COVID-19 Impact on Financial and Emotional Well-being*

|  | Wave 1 | | Wave 2 | |
| --- | --- | --- | --- | --- |
| Economic | % | n | % | n |
| Unable to get enough food or healthy food. | 6.45 | 32 | 2.22 | 11 |
| Unable to access clean water. | 0.20 | 1 | 0.20 | 1 |
| Unable to pay important bills like rent or utilities. | 3.63 | 18 | 3.83 | 19 |
| Difficulty getting places due to less access to public transportation or concerns about safety. | 13.71 | 68 | 13.91 | 69 |
| Unable to get needed medications (e.g., prescriptions or over-the-counter). | 6.45 | 32 | 2.42 | 12 |
| Emotional Health and Well-Being | % | n | % | n |
| Increase in child behavioral or emotional problems. | 2.42 | 12 | 1.61 | 8 |
| Increase in child’s sleep difficulties or nightmares. | 1.81 | 9 | 1.41 | 7 |
| Increase in mental health problems or symptoms (e.g., mood, anxiety, stress). | 72.58 | 360 | 62.70 | 311 |
| Increase in sleep problems or poor sleep quality. | 66.73 | 331 | 49.60 | 246 |
| Increase in use of alcohol or substances. | 35.08 | 174 | 23.79 | 118 |
| Unable to access mental health treatment or therapy. | 8.06 | 40 | 7.66 | 38 |
| Not satisfied with changes in mental health treatment or therapy. | 12.50 | 62 | 8.87 | 44 |
| Spent more time on screens and devices (e.g., looking at phone, playing video games, watching TV). | 94.15 | 467 | 83.87 | 416 |

*Note. n* = the number of participants who endorsed each variable. % = the percentage of participants that endorsed each variable.

**Table S6**

*COVID-19 Impact on Home Life*

|  | Wave 1 | | Wave 2 | |
| --- | --- | --- | --- | --- |
| Home Life | % | n | % | n |
| Childcare or babysitting unavailable when needed. | 2.82 | 14 | 3.23 | 16 |
| Difficulty taking care of children in the home. | 2.82 | 14 | 3.02 | 15 |
| More conflict with child or harsher in disciplining child or children. | 1.41 | 7 | 1.81 | 9 |
| Had to take over teaching or instructing a child. | 3.43 | 17 | 1.41 | 7 |
| Family or friends had to move into your home. | 4.64 | 23 | 2.22 | 11 |
| Had to spend a lot more time taking care of a family member. | 7.06 | 35 | 5.24 | 26 |
| Had to move or relocate. | 12.90 | 64 | 11.49 | 57 |
| Became homeless. | 0.00 | 0 | 0.20 | 1 |
| Increase in verbal arguments or conflict with a partner or spouse. | 21.98 | 109 | 18.15 | 90 |
| Increase in physical conflict with a partner or spouse. | 0.00 | 0 | 0.40 | 2 |
| Increase in verbal arguments or conflict with other adult(s) in home. | 10.28 | 51 | 6.45 | 32 |
| Increase in physical conflict with other adult(s) in home. | 0.20 | 1 | 1.01 | 5 |
| Increase in physical conflict among children in home. | 0.00 | 0 | 0.40 | 2 |
| Social Activities | % | n | % | n |
| Separated from family or close friends. | 81.45 | 404 | 54.03 | 268 |
| Did not have the ability or resources to talk to family or friends while separated. | 3.63 | 18 | 2.82 | 14 |
| Unable to visit loved one in a care facility (e.g., nursing home, group home). | 17.14 | 85 | 15.12 | 75 |
| Family celebrations cancelled or restricted. | 85.28 | 423 | 75.02 | 372 |
| Planned travel or vacations cancelled. | 90.12 | 447 | 75.62 | 375 |
| Religious or spiritual activities cancelled or restricted. | 32.06 | 159 | 24.19 | 120 |
| Unable to be with a close family member in critical condition. | 9.88 | 49 | 7.86 | 39 |
| Unable to attend in-person funeral or religious services for a family member or friend who died. | 12.90 | 64 | 14.92 | 74 |
| Unable to participate in social clubs, sports teams, or usual volunteer activities. | 62.90 | 312 | 53.63 | 266 |
| Unable to do enjoyable activities or hobbies. | 90.12 | 447 | 73.59 | 365 |

*Note. n* = the number of participants who endorsed each variable. % = the percentage of participants that endorsed each variable.
